# Macrophage pyroptosis mediates hyperoxia-induced inflammatory lung injury in neonates

**DOI:** 10.3389/fimmu.2025.1546986

**Published:** 2025-05-22

**Authors:** Zikun Tu, Haiyan Guo, Yajing Gao, Wenfeng Xiao, Xueru Xie, Hongmiao Yu, Qiuyan Liang, Yufeng Zhou

**Affiliations:** ^1^ Department of Critical Care Medicine, Children’s Hospital of Fudan University, National Children’s Medical Center, and the Shanghai Key Laboratory of Medical Epigenetics, International Co-laboratory of Medical Epigenetics and Metabolism, Ministry of Science and Technology, Institutes of Biomedical Sciences, Fudan University, Shanghai, China; ^2^ Department of Clinical Laboratory, Shanghai Ninth People’s Hospital, Shanghai Jiaotong University School of Medicine, Shanghai, China; ^3^ National Health Commission (NHC) Key Laboratory of Neonatal Diseases, Fudan University, Shanghai, China; ^4^ Fujian Key Laboratory of Neonatal Diseases, Xiamen Key Laboratory of Neonatal Diseases, Xiamen Children`s Hospital (Children’s Hospital of Fudan University at Xiamen), Xiamen, China

**Keywords:** bronchopulmonary dysplasia (BPD), macrophage, pyroptosis, gasdermin D (GSDMD), VX-765

## Abstract

**Background:**

Hyperoxia plays a key role in the development of bronchopulmonary dysplasia (BPD), a chronic lung disease of preterm infants. This study aimed to investigate the role of NLRP3/caspase-1/gasdermin D (GSDMD)-mediated pyroptosis in hyperoxia-induced lung injury in neonatal mice and to evaluate the potential protective effects of the caspase-1 inhibitor VX-765 on alveolar and vascular development in hyperoxia-exposed lungs.

**Materials and methods:**

C57/BL6 mouse pups were randomized on postnatal Day 4 (PN4) to receive daily intraperitoneal injections of VX-765, an effective and selective caspase-1 inhibitor, or a vehicle during exposure to room air or hyperoxia (85% O_2_) for 10 days. Alveolarization was assessed by H&E staining. Pulmonary vascular development was detected by CD31 immunohistochemistry. The degree of fibrosis was analyzed by Masson staining. TUNEL and Ki67 immunofluorescence staining was performed to assess overall cell survival in lung tissue. Concentrations of IL-1β was detected by ELISA in lung homogenates. The expressions of pyroptosis-associated proteins, NLRP3, Caspase-1 p20, N-GSDMD and mature IL-1β were evaluated by Western blot. Immunofluorescence colocalization of F4/80 with NLRP3/Caspase-1/IL-1β was performed. CD68 and AQP5 protein expression was analyzed by immunohistochemistry.

**Results:**

Hyperoxia activated the NLRP3 inflammasome, increased the production of mature IL-1β, and upregulated the expression of N-GSDMD, the active form of GSDMD that is responsible for the programmed cell death mechanism of pyroptosis in lung tissue. Importantly, VX-765 decreased NLRP3, IL-1β activation, and N-GSDMD expression and improved alveolar and vascular development by inhibiting pyroptosis of macrophages in hyperoxia-exposed lungs. Moreover, VX-765 also promoted cell proliferation and AT1 survival in the hyperoxia-exposed lung.

**Conclusion:**

NLRP3/Caspase-1/GSDMD-mediated pyroptosis plays a critical role in hyperoxia-induced neonatal lung injury, and targeting this pathway may be beneficial for the prevention of lung injury in preterm infants.

## 1 Introduction

Over 15 million infants are born preterm annually worldwide (1). Extremely premature infants, born at less than 28 weeks’ gestation, have a high risk of developing multi-organ injury and developmental abnormalities, predominantly affecting the lungs, brain, and eyes (2, 3). These premature infants, born with immature lungs, often experience respiratory failure shortly after birth and require oxygen (O_2_) therapy to survive. However, while high-concentration O_2_ therapy (hyperoxia) is life-saving, it can also cause lung inflammation that leads to bronchopulmonary dysplasia (BPD). BPD is characterized by disrupted alveolar and vascular development and reduced lung function (2). In the United States, treating BPD costs approximately $3 billion annually. Additionally, the mortality rate for patients with severe BPD complicated by pulmonary hypertension is as high as 50% (4). Despite recent advances in neonatal intensive care and extensive research, the pathogenesis of BPD in premature infants remains poorly understood, and no effective therapy has been identified. Thus, identifying new molecular pathways that impair alveolar growth and regeneration after injury of the immature lung may lead to potential novel therapeutics.

Pyroptosis, a novel form of pro-inflammatory cell death, has been implicated in various respiratory diseases, including idiopathic pulmonary fibrosis (IPF) (5), acute lung injury (ALI) (6), and asthma (7). This process is tightly regulated by inflammasomes, which are intracellular multiprotein complexes composed of nucleotide-binding domain leucine-rich repeat containing (NLR) proteins, the adaptor protein apoptosis-associated speck-like protein containing CARD (ASC), and the effector enzyme caspase-1 (8). Inflammasomes are activated by the innate immune system in response to various stimuli, including pathogen-associated molecular patterns (PAMPs) and host-derived danger-associated molecular patterns (DAMPs). In pyroptosis, the signaling cascade is initiated by NOD-like receptor pyrin domain-containing 3 (NLRP3) inflammasome assembly, typically triggered by PAMPs and DAMPs (9). Activated caspase-1 subsequently undergoes auto-proteolysis to generate active p20 and p10 subunits, which mediate two key events: cleavage of gasdermin D (GSDMD) to form membrane pores, and processing of pro-IL-1β and pro-IL-18 into mature cytokines (10, 11). Elevated levels of NLRP3 and its downstream effectors—such as caspase-1, GSDMD, and IL-1β—have been detected in the lungs of animal models of BPD. Notably, genetic deletion of NLRP3 or GSDMD alleviates inflammation and reduces vascular leakage in these models (12, 13). Through simultaneous induction of cell lysis and the release of pro-inflammatory cytokines, pyroptosis drives a vicious cycle of tissue damage and immune activation.

There is a growing recognition of the crucial role IL-1β plays in the inflammatory pathogenesis of BPD. Clinical studies have shown that increased levels of IL-1β in the tracheal aspirates of preterm infants correlate with a higher incidence of BPD (14, 15). Experimental models of BPD have also demonstrated that treatment with an IL-1 receptor antagonist can prevent lung inflammation and injury in newborn mice exposed to antenatal inflammation and moderate postnatal hyperoxia (16). These findings highlight the critical role of IL-1β in the pathogenesis of neonatal lung injury. While previous studies have primarily focused on upstream inflammasome components or downstream cytokine blockade (e.g., IL-1 receptor antagonists, NLRP3 knockout) (13), the direct targeting of caspase-1, the central effector protease, remains underexplored in neonatal lung disease. This gap is notable given the established safety profile of caspase-1 inhibitors in other inflammatory conditions and their potential advantage over broad-spectrum anti-inflammatory approaches that may compromise host defense. Therefore, we utilized VX-765, an effective and specific inhibitor of caspase-1 (17, 18), to investigate the mechanism and effects of pyroptosis on lung injury following hyperoxia exposure in newborn mice.

## 2 Materials and methods

### 2.1 Western blotting

Lungs were lysed with RIPA lysis buffer. SDS-PAGE was used to quantify total protein, which was then transferred to a polyvinylidene difluoride (PVDF) membrane (Millipore, Bedford, MA, USA) for immunoblotting using the antibodies listed below: anti-β-tubulin diluted 1:5000 (ab6046, Abcam, UK), anti-GSDMD diluted 1:1000 (ab209845, Abcam, UK), anti-NLRP3 diluted 1:1000 (AG-20B-0042, Adipogen, USA), anti-caspase1 (caspase-1 p20) diluted 1:1000 (AG-20B-0014, Adipogen, USA), anti-IL-1β diluted 1:1000 (12507, CST, USA), anti-VEGF diluted 1:1000 (sc-7269, Santa Cruz, USA), anti-VEGFR2 diluted 1:1000 (sc-6251, Santa Cruz, USA), anti-α-SMA diluted 1:1000 (sc-53142, Santa Cruz, USA), Rabbit Anti-Mouse IgG L(D3V2A) (HRP) diluted 1:5000 (58802, CST, USA), and Mouse Anti-Rabbit IgG L27A9 (HRP) diluted 1:5000 (5127, CST, USA). Proteins were visualized using enhanced chemiluminescence reagents (A38555, Thermo Scientific, USA) and were analyzed using a Bio-Rad ChemiDoc XRS + gel documentation system.

### 2.2 Animals

Male and female, 8–10-week-old C57BL/6 mice were obtained from Beijing Vital River Laboratory Animal Technology Co., Ltd. (under license number SCXK(Jing)2021-0006). Adult and neonatal mice were housed in a temperature- (22 ± 1°C) and humidity- (60 ± 5%) controlled room with a 12-hour light/dark rotation. Water and standard rodent chow were provided ad libitum. All experiments were authorized by the Animal Care and Use Committee at the Children’s Hospital of Fudan University (No. 2020-64), which complies with the Care and Use of Laboratory Animals guidelines of the National Institutes of Health (1978 revision, National Institutes of Health publication #8023).

### 2.3 Neonatal mouse model of BPD

Newborn mice were pooled and randomized to dams at the day of birth (born within 12 h of each other). Half of each litter was exposed to 85% O_2_ (hyperoxia, HO) for 7 or 14 days, whereas the other pups were exposed to room air (21% O_2_, RA) as described previously (19). The nursing dams were rotated between hyperoxia and Room Air litters every 24 h to avoid O_2_-related toxic effects on the dams. Exposure to hyperoxia was performed in a 90 × 42 × 38 cm plexiglas chamber. Oxygen concentrations were monitored with a Miniox II monitor (Hangzhou, China).

### 2.4 Treatment with VX-765

Newborn C57BL/6 mice were randomized to receive room air (21% O_2_) plus vehicle (normal saline), room air plus VX-765 (a potent and selective inhibitor of the IL-converting enzyme/caspase-1), hyperoxia (85% O_2_) plus vehicle, or hyperoxia plus VX-765 from Postnatal Day 4 (PN4) after being exposed to a hyperoxic environment. VX-765 (50 mg/kg, HY-13205, MedChemExpress, USA) or an equal volume of vehicle was administered daily using intraperitoneal injection during continuous exposure to room air or hyperoxia for 10 days. On PN14, the pups were anesthetized using 0.1% isoflurane, tracheotomized, and cannulized, and subsequently euthanized for analyses.

### 2.5 Histopathological analysis

Lungs were infused with 4% paraformaldehyde via a tracheal catheter at 25 cm H_2_O pressure for 5 min, fixed overnight, embedded in paraffin wax, and then sectioned. The lung samples were sliced into 5 μm paraffin sections. The sections were stained with a hematoxylin and eosin (H&E) staining kit (G1003, Servicebio, China) and a Masson’s trichrome (MASSON) staining kit (G1006, Servicebio, China) according to the manufacturer’s instructions. Immunohistochemical staining of paraffin sections was also performed to localize specific protein antigens in the lungs. Briefly, sections were subjected to antigen repair, endogenous enzyme quenching, and blocking with 5% goat serum. Primary antibodies anti-CD68 diluted 1:200 (GB113109-100, Servicebio, China), anti-CD31 diluted 1:200 (GB11063-2-100, Servicebio, China) and anti-AQP5 diluted 1:200 (GB113318-100, Servicebio, China) were incubated at 4 °C overnight.

After secondary antibodies were added, specific antigens were visualized with the DAB substrate kit (G1211, Servicebio, China), counterstained with hematoxylin, and sealed with neutral gum. All stained sections were microphotographed using a bright field microscope (Olympus BX53, Olympus, Japan).

### 2.6 Immunofluorescence assay

Lung sections were blocked with 5% goat serum and then incubated with the following primary antibodies: anti-Ki67 diluted 1:200 (GB121141-100, Servicebio, China), anti-NLRP3 diluted 1:200 (AG-20B-0042, Adipogen, USA), anti-caspase-1 (caspase-1 p20) diluted 1:200 (AG-20B-0014, Adipogen, USA), anti-GSDMD diluted 1:200 (ab209845, Abcam, UK) and anti-F4/80 diluted 1:200 (GB113373-100, Servicebio, China) overnight in a 4°C humidified chamber. Next, fluorescent secondary antibodies diluted 1:500 (G1222 and G1223, Servicebio, China) were added to each slide before counterstaining the section with DAPI (G1012, Servicebio, China). Finally, the slides were sealed with 50% glycerol. Images were captured using a Pannoramic MIDI slide scanner.

### 2.7 Immunofluorescence double-staining assay

Lung sections were dewaxed, antigen-repaired, and incubated with two primary antibodies: anti-caspase-1 (caspase-1 p20) diluted 1:200(AG-20B-0014, Adipogen, USA) and anti-F4/80 diluted 1:200(GB113373-100, Servicebio, China) followed by fluorescent secondary antibodies (G1222 and G1223, Servicebio, China). DAPI (G1012, Servicebio, China) was used to stain the sections, and images were captured using a Pannoramic MIDI slide scanner.

### 2.8 Lung Histology and Morphometry

Lungs were infused with 4% paraformaldehyde via a tracheal catheter at 25 cm H_2_O pressure for 5 min, fixed overnight, embedded in paraffin wax, and then sectioned. Hematoxylin and eosin staining was performed for lung histology, radial alveolar count (RAC), and mean linear intercept (MLI) measurements as previously described (20). Micro-vessel density (MVD) assesses the degree of microvascular development by CD31 staining in lung tissues (21).

### 2.9 Assessment of lung inflammation

Macrophage infiltration was determined by immunostaining with an anti-CD68 antibody. The number of CD68-stained cells in the alveolar airspaces of lung tissue sections were counted from five random high power fields (HPF) taken from the 20 × objective on each slide.

### 2.10 Terminal transferase dUTP nick-end labeling

*In situ* TUNEL for the detection of apoptosis was performed on PN14 tissue sections using an *in situ* cell death detection fluorescence kit (G1504-50T, Servicebio, China) as the per manufacturer’s instructions. TUNEL-positive cells were counted in at least 10 nonoverlapping HPFs (×400 magnification) from three to four animals per group.

### 2.11 Assessment of lung cell proliferation and death

Cell proliferation was assessed by immunofluorescent staining for Ki67, and the proliferating index was calculated as the average percentage of Ki67-positive nuclei in total nuclei in five random HPF on lung sections from each animal. Cell death was studied using a TUNEL assay and the cell death index was calculated as the average percentage of TUNEL-positive nuclei in total nuclei in five random HPF on lung sections from each animal (22).

### 2.12 IL-1β ELISA

Lung tissue homogenate was used for ELISA to determine the IL-1β concentration. ELISA was performed according to the manufacturer’s guidance (IL-1β Mouse Uncoated ELISA Kit, Invitrogen, #88-7013-22, USA).

### 2.13 ROS staining of frozen section experiment

Lung tissue frozen sections were stained with ROS dye solution (Sigma Aldrich, D7008, USA) to detect ROS level according to the manufacturer’s instructions.

### 2.14 Statistical data analysis

Image Pro Plus 6.0 and Image J software were used for quantitative analysis of histological sections and western blotting. All data are given as mean value ± SEM. Statistically significant differences between different groups were investigated using one-way analysis of variance (ANOVA) with Prism 9.0 software (GraphPad Software, Inc.) supplemented with Student’s t-test (ns, not significant, * P < 0.05, ** P < 0.01, *** P < 0.001, **** P < 0.0001). Tukey’s multiple comparison test was also used. For data in Gaussian distribution and without homogeneity variance, Welch’s correction was used.

## 3 Results

### 3.1 Hyperoxia impaired alveolar tissue and vascular growth in neonatal mice

**Figure 1:**
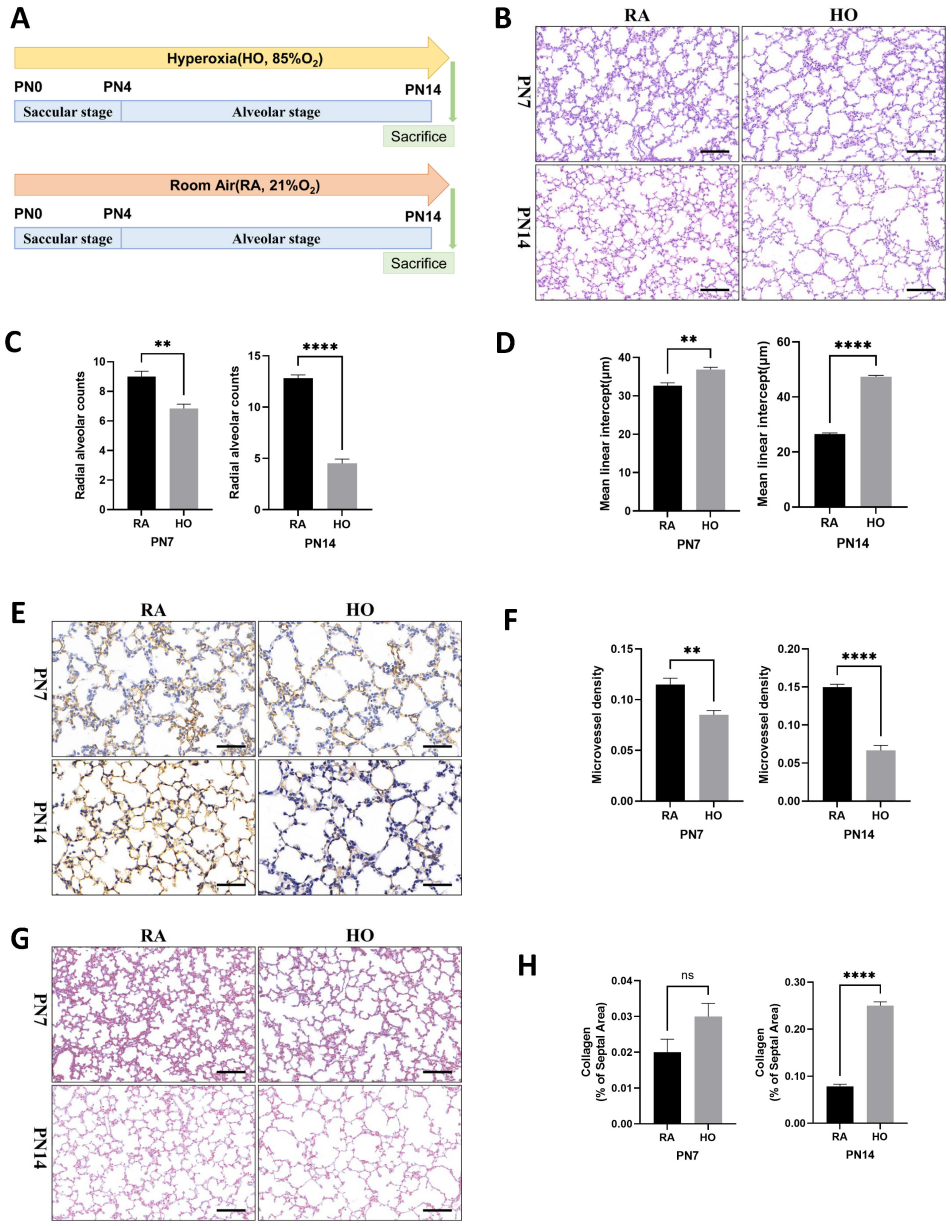
Hyperoxia impaired alveolar tissue and vascular growth in neonatal mice. **(A)** Schematic representation of mouse model of hyperoxia-induced bronchopulmonary dysplasia (BPD). Newborn mice were exposed to room air (21% O_2_, RA) or hyperoxia (85% O_2_, HO) from postnatal Day 1 (PN1) to postnatal Day 14 (PN14). Mice were sacrificed on PN14. **(B)** Representative H&E staining lung tissue sections from PN7-RA, PN7-HO, PN14-RA, and PN14-HO mice (scale bar = 50 μm). **(C)** Quantitative analysis of radial alveolar count (RAC) analyzed using Image J on H&E staining. **(D)** Quantitative analysis of mean linear intercept (MLI) analyzed using Image J on H&E staining. **(E)** IHC staining of CD31 (marker of endothelial cells, yellow-brown area) expression in the lungs of pups on PN7 and PN14 (scale bar = 20 μm). **(F)** Quantitative analysis of mean micro-vessel density (MVD) measured using Image-Pro Plus 6.0 on IHC staining of CD31. **(G)** Masson staining in the lungs of pups on PN7 and PN14 (scale bar = 50 μm). **(H)** Quantitative analysis of collagen of the septal area analyzed using Image J on masson staining. Data represent results from three individual studies. Statistical analysis of the data was performed using Student’s t test **(C, D, F, H)**. Data are shown as mean ± SEM; n = 6 per group. ns, not significant, **P<0.01, ****P<0.0001. H&E, hematoxylin and eosin; IHC, immunohistochemistry.

Exposure to hyperoxia led to lung impairment on postnatal Day 7 (PN7) and postnatal Day 14 (PN14), characterized by a heterogeneous distribution of enlarged air spaces, reduced radial alveolar count (RAC), and increased mean linear intercept (MLI) at both time points (**Figures 1A–D**). CD31 immunohistochemistry staining revealed that exposure to hyperoxia significantly suppressed vascular development in the lung of pups, resulting in reduced mean micro-vessel density (MVD) on both PN7 and PN14 (**Figures 1E, F**). Masson staining showed no difference in collagen deposition between the control and hyperoxia group on PN7, but a significant increase in collagen deposition was observed on PN14 (**Figures 1G, H**), suggesting that lung tissue fibrosis mainly occurs in the late stage of BPD. These results were consistent with the pathological hallmarks of BPD, indicating that hyperoxia impaired the growth of alveolar and vascular tissues in neonatal mice.

### 3.2 Cell survival decreased and cell death increased in the hyperoxia-exposed lungs

**Figure 2:**
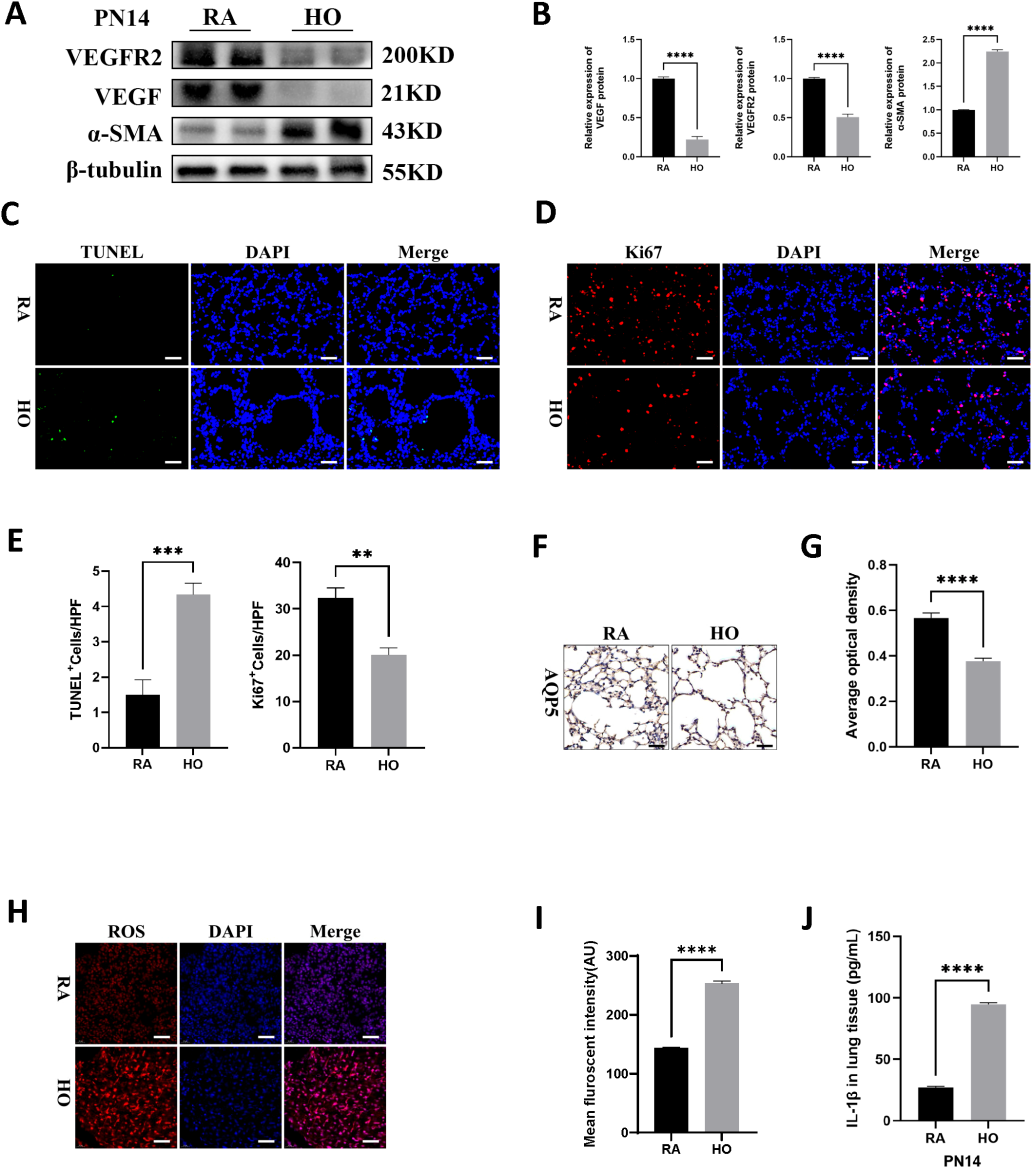
Cell survival decreased and cell death increased in hyperoxia-exposed lungs. **(A)** Western blots of VEGFR2, VEGF, and α-SMA expression in hyperoxic lungs on postnatal Day 14 (PN14). **(B)** Quantification of protein expression on **(A)** measured using Image J. **(C)** TUNEL Assay (green signals) and DAPI nuclear stain (blue signals) were used to identify dead cells on PN14 (scale bar = 20 μm). **(D)** IF staining for Ki67 (red signals) and DAPI staining (blue signals) were performed to assess cell proliferation on PN14 (scale bar = 20 μm). **(E)** Quantification of cell death (percentage of apoptotic nuclei divided by total nuclei) and the cell proliferation index (percentage of Ki67 positive nuclei divided by total nuclei). **(F)** IHC staining for AQP5 (yellow-brown area) was performed to evaluate the survival of alveolar epithelial cells type I (ATI) on PN14 (scale bar = 20 μm). **(G)** Quantitative analysis of the AQP5-positive area analyzed using Image J. **(H)** IF staining for reactive oxygen species (ROS) (red signals) was performed to assess the redox level in lung on PN14 (scale bar = 20 μm). **(I)** Quantitative analysis of the mean fluorescence intensity of ROS analyzed using Image J. **(J)** IL-1β was detected using ELISA in the lungs of pups on PN14. Data represent results from three individual studies. Statistical analysis of the data was performed using Student’s t test **(B, E, G, I, J)**. Data are shown as mean ± SEM; n = 6 per group. **P < 0.01, ***P < 0.001, ****P < 0.000. IHC, immunohistochemistry; IF, immunofluorescent.

Impaired vascularization and pulmonary vascular remodeling are key features of hyperoxia-induced lung injury and are associated with the development of pulmonary hypertension. Compared to the room air group, the hyperoxia group exhibited lower expression levels of vascular endothelial growth factor (VEGF) and VEGF receptor 2 (VEGFR2), but had higher protein levels of α-smooth muscle actin (α-SMA), a pulmonary fibrosis biomarker on PN14 (**Figures 2A, B**). Immunofluorescence staining showed that hyperoxia significantly suppressed cell proliferation and increased cell death in the lungs of pups (**Figures 2C–E**). Additionally, hyperoxia reduced the expression of aquaporin 5 (AQP5), an important marker of type I alveolar epithelial cells (ATI), indicating severe impairment of ATI in hyperoxia-stimulated lungs (**Figures 2F, G**). Furthermore, the levels of reactive oxygen species (ROS) were significantly elevated in hyperoxia-exposed lungs (**Figures 2H, I**). We also found that hyperoxia significantly increased the production of mature IL-1β in the lungs of pups on PN14 (**Figure 2J**). This indicates that sustained hyperoxic stimulation creates a hyperinflammatory environment in the lung tissue of BPD mice.

### 3.3 NLRP3/Caspase-1/GSDMD-mediated macrophage pyroptosis was significantly enhanced in bronchopulmonary dysplasia

**Figure 3:**
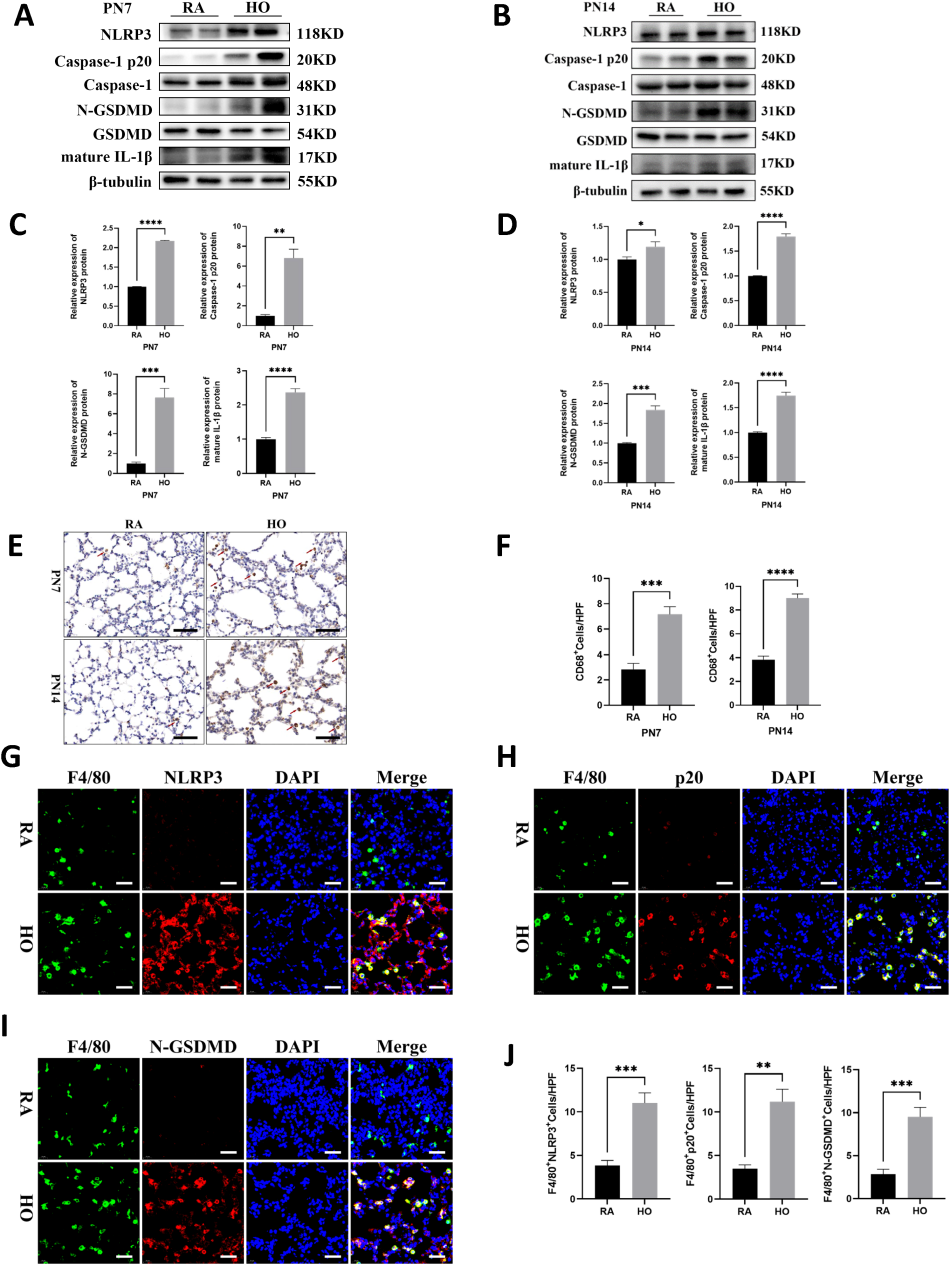
NLRP3/Caspase-1/GSDMD-mediated macrophage pyroptosis was significantly enhanced in bronchopulmonary dysplasia (BPD). **(A, B)** Western blots of pyroptosis-related proteins expression in the hyperoxic lung on postnatal Day 7 (PN7) and postnatal Day 14 (PN14). **(C, D)** Quantification of protein expression in Figure **(A, B)** measured using Image J. **(E)** IHC staining for CD68 (marker of macrophages, indicated with red arrows) in the lungs of pups on PN7 and PN14 (scale bar = 20 μm). **(F)** Quantitative analysis of the CD68-positive area analyzed using Image J. **(G)** IF co-localization images of F4/80 (marker of mouse macrophages, green) with NLRP3 (red) in lung tissues on PN14, DAPI (blue) (scale bar=20 μm). **(H)** IF co-localization images of F4/80 (green) with Capase-1 p20 (red) in lung tissues on PN14, DAPI (blue) (scale bar=20 μm). **(I)** IF co-localization images of F4/80 (green) with N-GSDMD (red) in lung tissues on PN14, DAPI (blue) (scale bar=20 μm). **(J)** Quantitative analysis of double-positive cells on Figure **(G-I)**. Data represent results from three individual studies. Statistical analysis of the data was performed using Student’s t test **(C, D, F, J)**. Data are shown as mean ± SEM; n = 6 per group. *P < 0.05, **P < 0.01, ***P < 0.001, ****P < 0.000. IHC, immunohistochemistry; IF, immunofluorescent.

Western blot analysis revealed significant activation of NLRP3 inflammasome, significant increases in Caspase-1 p20 expression, N-GSDMD cleavage, and the secretion of mature IL-1β in the hyperoxia group on PN7 and PN14; in contrast, these markers were nearly undetectable in the air control group (**Figures 3A-D**). Histological examination showed a marked infiltration of macrophages in the hyperoxia-exposed lungs on PN7 and PN14 compared to those of the control group (**Figures 3E, F**). Additionally, we performed double fluorescent staining on lung sections to evaluate co-localization of NLRP3, Caspase-1 p20, and N-GSDMD (shown in red) with F4/80 (shown in green) on PN14. The hyperoxia group exhibited the brightest red and green fluorescence, and importantly, the fluorescence merged completely (yellow color) in the hyperoxia group (**Figures 3G-J**); whereas, the co-localized yellow color was significantly weaker in the room air group. These findings indicated that the canonical signaling pathway of pyroptosis in macrophages was markedly activated after exposure to hyperoxia.

### 3.4 Caspase-1-specific inhibitor VX-765 improved alveolar development in the mice of hyperoxia-induced bronchopulmonary dysplasia

**Figure 4:**
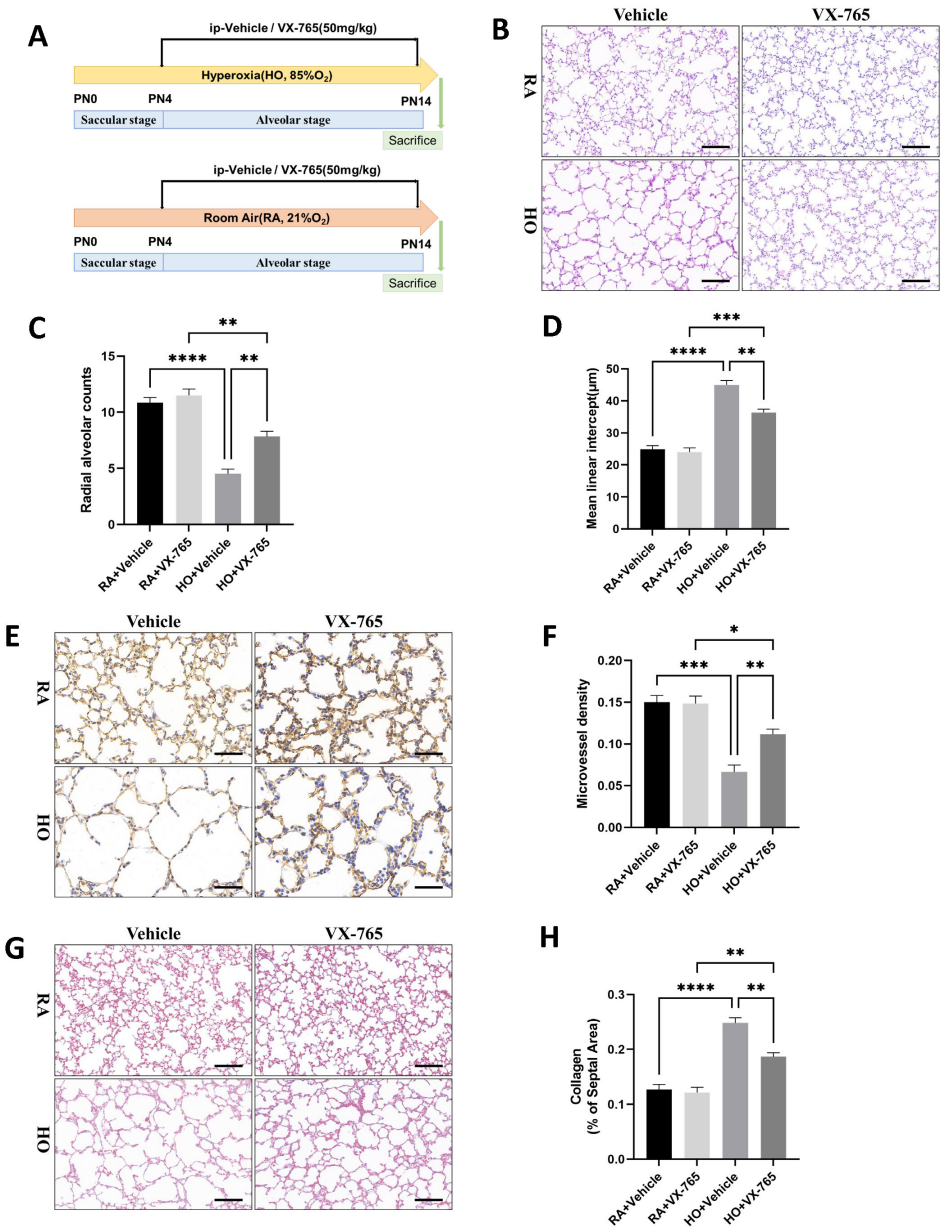
Caspase-1-specific inhibitor VX-765 improved alveolar development in the mice of hyperoxia-induced bronchopulmonary dysplasia (BPD). **(A)** Schematic representation of mouse model of hyperoxia-induced BPD with VX-765 treatment. Newborn mice were randomized to receive room air (21% O_2_) plus vehicle (saline), room air plus VX-765, hyperoxia (85% O_2_) plus vehicle, or hyperoxia plus VX-765 from postnatal Day 4 (PN4) after being exposed to a hyperoxic environment. Mice were sacrificed on postnatal Day 14 (PN14). **(B)** Representative H&E staining lung tissue sections from each group on PN14 (scale bar = 50 μm). **(C)** Quantitative analysis of radial alveolar count (RAC) analyzed using Image J on H&E staining. **(D)** Quantitative analysis of mean linear intercept (MLI) analyzed using Image J on H&E staining. **(E)** IHC staining of CD31 (marker of endothelial cells, yellow-brown area) expression in the lungs of pups from each group on PN14 (scale bar = 20 μm). **(F)** Quantitative analysis of mean micro-vessel density (MVD) measured using Image-Pro Plus 6.0 on IHC staining of CD31. **(G)** Masson staining in the lungs of pups from each group on PN14 (scale bar = 50 μm). **(H)** Quantitative analysis of collagen of the septal area analyzed using Image J on masson staining. Data represent results from three individual studies. Statistical analysis of the data was performed using one-way ANOVA **(C, D, F, H)**, followed by Tukey’s multiple comparisons test. Data are shown as mean ± SEM; n = 6 per group. *P < 0.05, **P < 0.01, ***P < 0.001, ****P < 0.0001. H&E, hematoxylin and eosin; IHC, immunohistochemistry.

**Figure 5:**
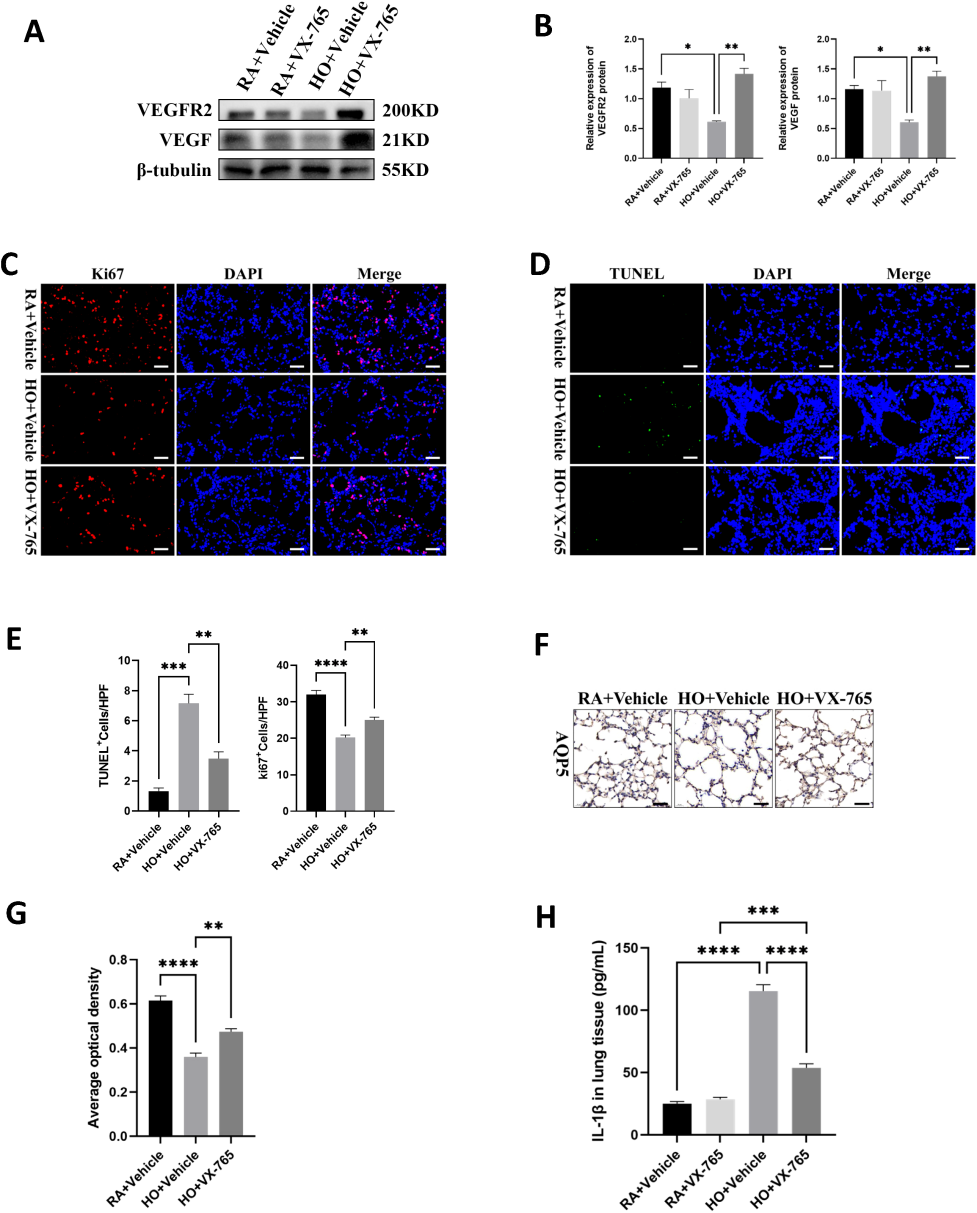
VX-765 enhanced cell survival and reduced cell death in the mice lung tissue of bronchopulmonary dysplasia (BPD). **(A)** Western blots of VEGFR2, VEGF expression in the lungs of mice from each group on postnatal Day 14 (PN14). **(B)** Quantification of protein expression on **(A)** measured using Image J. **(C)** IF staining for Ki67 (red signals) and DAPI staining (blue signals) were performed to assess cell proliferation on PN14 (scale bar = 20 μm). **(D)** TUNEL Assay (green signals) and DAPI nuclear stain (blue signals) were used to identify dead cells on PN14 (scale bar = 20 μm). **(E)** Quantification of cell death (percentage of apoptotic nuclei divided by total nuclei) and the cell proliferation index (percentage of Ki67 positive nuclei divided by total nuclei). **(F)** IHC staining for AQP5 (yellow-brown area) was performed to evaluate the survival of alveolar epithelial cells type I (ATI) on PN14 (scale bar = 20 μm). **(G)** Quantitative analysis of the AQP5-positive area analyzed using Image J. **(H)** IL-1β was detected by ELISA in the lung of pups from each group on PN14. Data represent results from three individual studies. Statistical analysis of the data was performed using one-way ANOVA **(B, E, G, H)**, followed by Tukey’s multiple comparisons test. Data are shown as mean ± SEM; n = 6 per group. *P < 0.05, **P < 0.01, ***P < 0.001, ****P < 0.0001. IHC, immunohistochemistry; IF, immunofluorescent.

We investigated the effects of caspase-1 inhibition on alveolar development (**Figure 4A**). As shown in **Figure 4B**, there was no difference in the degree of alveolarization between the groups exposed to room air. However, histologically, hyperoxia-exposed animals showed marked simplification of the alveoli, evidenced by larger alveoli with increased alveolar diameters, whereas treatment with VX-765 increased alveolarization as evidenced by decreased simplification. VX-765 administration increased RAC and decreased the MLI in treated hyperoxia-exposed lungs, indicating improved alveolarization compared with hyperoxia-exposed, vehicle-treated lungs (**Figures 4C, D**). Additionally, treatment with VX-765 significantly increased MVD (**Figures 4E, F**) and reduced collagen deposition (**Figures 4G, H**) during hyperoxia, compared with the hyperoxia-exposed, vehicle-treated group. Consistent with increased vascular development, VX-765 also upregulated the expression of VEGF as well as VEGFR2 and downregulated the expression of α-SMA (**Figures 5A, B**). These results indicated that the inhibition of caspase-1 improves angiogenesis and decreases vascular remodeling during hyperoxia exposure in newborn mice.

### 3.5 VX-765 enhanced cell survival and reduced cell death in the mice lung tissue of bronchopulmonary dysplasia

As previously stated, hyperoxia is known to reduce cell survival and cause cell death in BPD models. We observed that the hyperoxia-exposed, vehicle-treated group showed a distinct reduction in cell proliferation compared to the RA-exposed, vehicle-treated group (**Figures 5C, E**). However, the VX-765-treated hyperoxic group exhibited markedly increased cell proliferation compared to the vehicle-treated hyperoxic group (**Figures 5C, E**). When assessing cell death, the results showed that the hyperoxia-exposed, vehicle-treated group had a significant increase in cell death compared to the RA-exposed, vehicle-treated group (**Figures 5D, E**). In contrast, the hyperoxia-exposed group treated with VX-765 had significantly less cell death than the vehicle hyperoxic group (**Figures 5D, E**). Furthermore, VX-765 treatment greatly increased AQP5 expression and promoted AT1 cell survival in hyperoxia-stimulated lungs, thereby exerting a protective effect on the alveolar epithelium (**Figures 5F, G**). Additionally, VX-765 administration substantially reduced the secretion of mature IL-1β in hyperoxic lungs (**Figure 5H**).

### 3.6 VX-765 inhibited NLRP3/Caspase-1/GSDMD-mediated macrophage pyroptosis in the mice lung tissue of bronchopulmonary dysplasia (BPD).

**Figure 6:**
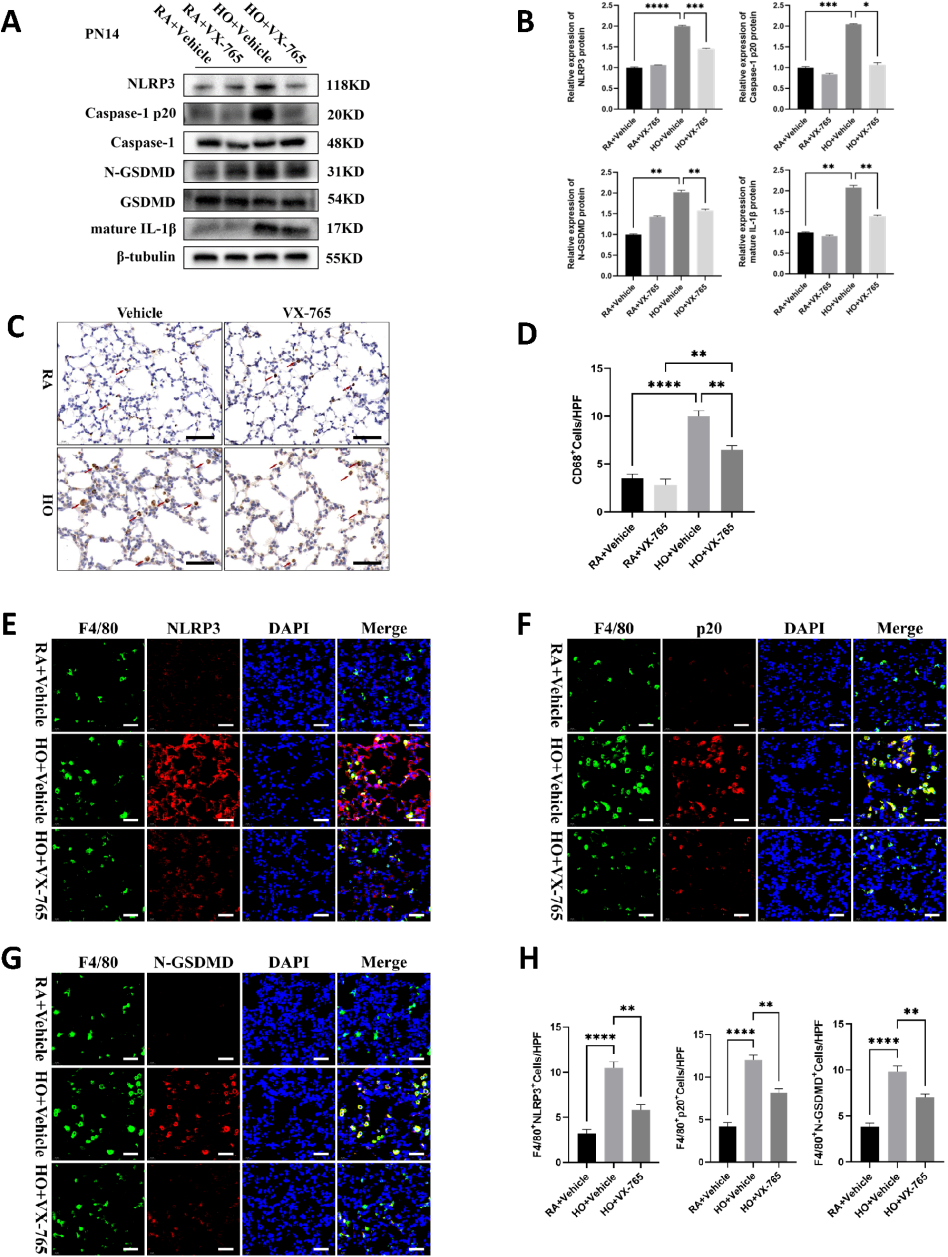
VX-765 inhibited NLRP3/Caspase-1/GSDMD-mediated macrophage pyroptosis in the mice lung tissue of bronchopulmonary dysplasia (BPD). **(A)** Western blots of pyroptosis-related protein expression in the lung from each group on postnatal Day 14 (PN14). **(B)** Quantification of protein expression on the Figure **(A)** measured using Image J. **(C)** IHC staining for CD68 (marker of macrophages, indicated with red arrows) in the lungs of pups from each group on PN14 (scale bar = 20 μm). **(D)** Quantitative analysis of the CD68-positive area analyzed using Image J. **(E)** IF co-localization images of F4/80 (marker of mouse macrophages, green) with NLRP3 (red) in lung from each group on PN14, DAPI (blue) (scale bar=20 μm). **(F)** IF co-localization images of F4/80 (green) with Capase-1 p20 (red) in lung from each group on PN14, DAPI (blue) (scale bar=20 μm). **(G)** IF co-localization images of F4/80 (green) with N-GSDMD (red) in lung from each group on PN14, DAPI (blue) (scale bar=20 μm). **(H)** Quantitative analysis of double-positive cells on Figure **(E-G)**. Data represent results from three individual studies. Statistical analysis of the data was performed using one-way ANOVA **(B, D, H)**, followed by Tukey’s multiple comparisons test. Data are shown as mean ± SEM; n = 6 per group. *P < 0.05, **P < 0.01, ***P < 0.001, ****P < 0.0001. IHC, immunohistochemistry; IF, Immunofluorescent.

We examined the effect of VX-765 administration on caspase-1 activity and the expression of NLRP3 inflammasome signaling proteins, along with pyroptosis-related proteins. Hyperoxia increased the expression of NLRP3, active Caspase-1 p20, and N-GSDMD in the vehicle-treated mouse pups, but these levels were significantly decreased in VX-765-treated mice (**Figures 6A, B**). Caspase-1 inhibition also significantly reduced the maturation and secretion of IL-1β (**Figures 5H**, **6A, B**). The hyperoxia-exposed, vehicle-treated mouse pups had a large increase in macrophage counts compared to the RA-exposed, vehicle-treated group (**Figures 6C, D**). However, VX-765 administration markedly decreased macrophage infiltration induced by exposure to hyperoxia (**Figures 6C, D**). Furthermore, immunofluorescence staining of lung tissue sections showed increased expression of NLRP3, Caspase-1 p20, and N-GSDMD in vehicle-treated mouse pups exposed to hyperoxia. Treatment with VX-765 also reduced the enhanced expression of these proteins in lung macrophages (**Figures 6E-H**).

## 4 Discussion

Bronchopulmonary dysplasia (BPD) is a leading cause of chronic lung disease in preterm infants, contributing significantly to long-term respiratory morbidity and mortality. Understanding the underlying mechanisms and developing effective therapies are critical for improving outcomes in this vulnerable population. Hyperoxia-induced neonatal lung injury models in newborn rodents are widely used for mechanistic studies and exploring potential therapies because they share many developmental similarities with preterm infants at risk for BPD (23, 24). In this study, we used a newborn mouse model to test the hypothesis that hyperoxia-induced lung injury is mediated by inflammasome activation and that inhibition of caspase-1, a critical component of the inflammasome cascade, attenuates hyperoxia-induced lung injury in neonatal mice. We observed that hyperoxia activated the NLRP3 inflammasome, increased macrophage infiltration, and decreased alveolarization and vascular development as well as pulmonary fibrosis in the neonatal lung. More importantly, we demonstrated that caspase-1 inhibition with VX-765, a potent bioavailable and nontoxic small molecule inhibitor of caspase‐1 (25), downregulated the NLRP3 inflammasome, reduced lung inflammation, decreased pyroptosis-mediated cell death, increased cell proliferation, improved alveolarization and vascular development, and reduced pulmonary fibrosis in hyperoxia-exposed mice. These results suggest that hyperoxia-induced lung injury may be mediated by the NLRP3-caspase-1-GSDMD axis, and targeting the inflammasome may be beneficial for preventing lung injury in preterm infants.

Macrophages play key roles in maintaining tissue homeostasis by initiating and resolving inflammation in the immune system (26). Clinical and animal studies have demonstrated increased neutrophils and macrophages in bronchoalveolar lavage fluid (27, 28) and tracheal aspirates from subjects with BPD (15) In this study, we found that antagonism of caspase-1 downregulated the expression of NLRP3 inflammasome proteins, leading to decreased macrophage infiltration in the alveolar spaces of lungs exposed to hyperoxia. This finding aligns with those of previous research showing that caspase-1-deficient mice exhibit markedly reduced leukocyte infiltration in the airway after rhinovirus infection (29). Recent findings have shown (30) that hyperoxia activates immature macrophages in the lungs, promotes the release of inflammatory cytokines, and damages regenerative sites in alveolar epithelial cells via the IL-6/STAT3 axis, thereby inhibiting lung growth and development. However, the mechanism by which hyperoxia leads to the massive release of inflammatory cytokines from macrophages is not fully understood. Based on the activation of NLRP3-mediated pyroptosis in BPD lung tissues, we further determined the occurrence of pyroptosis in massively infiltrated macrophages in the lung tissues of BPD mice using immunofluorescence co-localization of F4/80 with NLRP3, caspase-1 p20, and N-GSDMD. Pyroptosis further amplifies the lungs’ inflammatory immune response, resulting in epithelial and endothelial injury. As a result, lung macrophages play a crucial role in developing BPD in the early stages of inflammation and subsequent impaired alveolarization.

In contrast to previous studies, Fredrick et al. identified the presence of hyperactivated NLRP1-mediated pyroptosis in lung tissues of BPD rats (31), but our study also found that the cells undergoing NLRP3-mediated pyroptosis may be mainly inflammatory macrophages that are heavily infiltrated in the lungs. In addition, we found that NLRP3/Caspase-1/GSDMD mediated pyroptosis was also significantly activated in the 7-day hyperoxia group, suggesting that inflammation and pyroptosis are involved in the development of the disease at an early stage. However, collagen fibers were not significantly overexpressed until day 14, suggesting that inflammation and pyroptosis occur earlier than pulmonary fibrosis, and may be an important etiological factor in exacerbating the long-term pulmonary fibrosis and severe vascular dysplasia in BPD mice.

Although we demonstrated that hyperoxia induced macrophage pyroptosis to be activated in lung tissues of BPD mice, we still cannot exclude the possibility that other non-immune cells, such as epithelial or endothelial cells, also undergo pyroptosis. Moreover, pyroptosis is mainly an innate immune response. Macrophages are a central component of the intrinsic immune system, and it has also been shown that lung macrophages are essential for immunoregulatory functions in BPD (26, 32, 33). Therefore, we may be more inclined to consider that pyroptosis in intrinsic immune cells (i.e., macrophage) play a more dominant role in the pathogenesis of BPD than parenchymal cells in the lungs. Neutrophils are also an important component of the intrinsic immune system and are also involved in the inflammatory response in the BPD lungs (28); however, most studies on GSDMD-induced pyroptosis have focused on macrophages. Although GSDMD has been shown to have a role in neutrophil function, in contrast to macrophages, GSDMD activated by NLRP3 in neutrophils does not readily lead to pyroptosis (34), but rather promotes neutrophil extracellular trap (NET) formation by targeting nucleus formation (35). Recently, it has also been reported that neutrophils can indeed promote lung injury in BPD through NET (36, 37).

The deleterious effects of hyperoxia result from both direct injury mediated by reactive oxygen species (ROS) and indirect injury from lung inflammation (38). Hyperoxia causes cell injury because cellular antioxidant defenses become overwhelmed, leading to the accumulation of toxic levels of ROS and subsequently apoptosis of alveolar epithelial cells (39). Similarly, we found that ROS activity was significantly enhanced in hyperoxia-stimulated lungs, which is consistent with the findings of previous reports (40). The mechanism linking hyperoxia to the upregulation of caspase-1 in the pathogenesis of BPD remains unknown. Recent research indicates that mitochondrial ROS production is situated upstream of the NLRP3 inflammasome (41, 42) and that ROS can serve as a redox signaling molecule to activate the NLRP3 inflammasome (43). Furthermore, ROS blockade via chemical scavengers of ROS, as well as pharmacological and genetic inhibition of NADPH oxidase, have been shown to suppress inflammasome activation and pyroptosis in response to a wide range of stimuli (44, 45). Collectively, our results support the finding that NLRP3 inflammasome-mediated pyroptosis plays a key role in the pathogenesis of hyperoxia-induced lung injury.

BPD is characterized by alveolar simplification with “dysmorphic” microvasculature and decreased vessel growth (2, 24, 46). Among inflammatory mediators, IL-1 has emerged as a promising therapeutic target in neonatal inflammatory lung diseases. This is supported by evidence showing that treatment with an interleukin-1 receptor antagonist (IL-1Ra) mitigates murine BPD induced by perinatal inflammation and hyperoxia (47–49). VX-765, a peptidomimetic caspase-1 inhibitor, selectively blocks the cleavage of pro-IL-1β, thereby reducing IL-1β production during pyroptosis (50, 51). Although VX-765 has not previously been evaluated in experimental models of BPD, our study demonstrates, for the first time, its dual therapeutic potential in attenuating hyperoxia-induced pulmonary inflammation and preserving alveolar architecture. Specifically, pharmacological inhibition of caspase-1 signaling with VX-765 significantly improved alveolarization following 14 days of hyperoxia exposure. Further, we found improved vascular density accompanied by improved alveolarization in the VX-765-treated hyperoxia pups. Moreover, these structural changes were associated with increased expression of VEGF and VEGFR2. VEGF signaling is impaired in rodents exposed to chronic hyperoxia (52). The disruption of pulmonary VEGF leads to abnormal vascular and alveolar development and lung hypoplasia (53, 54). Previous studies have reported that caspase-1 activation impairs VEGFR2 expression and caspase-1 depletion improves angiogenesis (55, 56). Therefore, we postulate that the protective effects of VX-765 in alveolar and vascular development are due to its anti-inflammatory effects and ability to augment angiogenesis via caspase-1 inhibition. Our study has some limitations. First, we used extremely high levels of oxygen to induce lung injury, which is rarely used clinically in preterm infants. However, lung injury induced by this level of oxygen was like clinically severe BPD. Second, the etiology of lung injury in preterm infants is multifaceted, and we did not assess other contributing factors such as intrauterine infection and mechanical ventilation. It will be important to further investigate the role of caspase-1-mediated pyroptosis in intrauterine infection, postnatal moderate hyperoxia-induced injury, and mechanical ventilation-induced lung injury.

VX-765 was also well tolerated, as we did not observe any deleterious effects of caspase-1 inhibition in the lungs of room-air-exposed animals. Furthermore, VX‐765 has been proven to be safe for humans when administered orally in a 6-week-long phase II clinical trial that studied epilepsy (57). VX-765 has also been shown to alleviate Alzheimer’s disease and cardiovascular disease in animal models (58). Additionally, VX-765 has already advanced to clinical trials (NCT01048255, NCT00205465, NCT05164120), underlining its translational potential. For the first time, our animal experiments demonstrated that VX-765 alleviated tissue injury and effectively treated BPD in preclinical models by inhibiting caspase-1, thereby blocking the maturation of proinflammatory cytokines. Unlike broad-spectrum anti-inflammatory agents such as glucocorticoids, VX-765 selectively targets caspase-1, potentially reducing immunosuppressive side effects (e.g., infection risk). Therefore, VX-765 appears to be a promising therapeutic candidate warranting rapid translation into clinical trials for BPD patients. However, additional studies in neonatal animals are needed to assess the pharmacokinetics and effects on other developing organs before it can be used in clinical trials as a novel strategy to prevent and treat BPD in preterm infants. Therefore, we hope to further investigate the specific mechanism of inflammatory lung injury caused by the inflammatory death of macrophage pyroptosis in the early stages of hyperoxia exposure. While our study focused on the role of pyroptosis and inflammasome activation in BPD pathogenesis, it is important to acknowledge that BPD development involves multiple interconnected pathways beyond oxidative stress and inflammation. Emerging evidence highlights several critical mechanisms that contribute to impaired alveolarization and vascular dysregulation in preterm lungs, for example, dysregulated growth factor signaling (59), mitochondrial dysfunction (60) and microbiome dysbiosis (61). Future investigations should aim to elucidating these connections, which could offer deeper insights into BPD pathogenesis and inform combination therapeutic strategies.

## 5 Conclusions

In summary, we demonstrated that macrophage pyroptosis occurs in the early stages of BPD, and VX-765 can alleviate the development of BPD by inhibiting the pyroptosis signaling pathway. Inhibition of NLRP3/Caspase-1/GSDMD-mediated pyroptosis largely reversed the injurious effects of hyperoxia, resulting in attenuated lung inflammation, improved alveolarization and pulmonary vascular development, and alleviated pulmonary fibrosis.

## Data Availability

The original contributions presented in the study are included in the article/supplementary material. Further inquiries can be directed to the corresponding author.
